# Rescue levodopa‐carbidopa intestinal gel (LCIG) therapy in Parkinson’s disease patients with suboptimal response to deep brain stimulation

**DOI:** 10.1002/acn3.50889

**Published:** 2019-09-13

**Authors:** Ahmad Elkouzi, Adolfo Ramirez‐Zamora, Pam Zeilman, Matthew Barabas, Robert S. Eisinger, Irene A. Malaty, Michael S. Okun, Leonardo Almeida

**Affiliations:** ^1^ Fixel Institute for Neurological Diseases University of Florida Gainesville Florida

## Abstract

**Objective:**

To evaluate the effectiveness of levodopa‐carbidopa intestinal gel (LCIG) as an add‐on rescue therapy following deep brain stimulation (DBS) for treatment of motor fluctuations.

**Background:**

Both DBS and LCIG are FDA‐approved therapies for treatment of motor fluctuations in advanced PD. Few studies have examined dual therapy for refractory motor fluctuations and it is unknown what the effect on quality of life will be in advanced PD.

**Methods:**

We conducted a retrospective study using a large database of all medical and surgical PD cases at the University of Florida. Six patients were identified with DBS who subsequently received rescue LCIG therapy. The clinical histories, indications for intervention and outcomes were reviewed.

**Results:**

All patients were managed initially with DBS (bilateral STN DBS (*n* = 3), bilateral GPi DBS (*n* = 1), unilateral GPI DBS (*n* = 2)). Patients with well‐placed (*n* = 3) and suboptimally placed DBS leads (*n* = 3) had significant reduction in their motor fluctuations with improvement in the off‐medication time after rescue LCIG therapy. Improvement in quality of life scores (PDQ‐39) was appreciated in four DBS patients following the addition of LCIG therapy.

**Conclusions:**

LCIG is a promising add‐on rescue therapy for select patients with existing DBS devices. The LCIG may possibly reduce motor fluctuations and improve quality of life in advanced PD irrespective of the DBS target or the accuracy of lead placement. Dual therapy may also be ideal for patients who are considered high risk for additional DBS surgeries.

## Introduction

Motor fluctuations manifesting as off‐time and dyskinesia impair quality of life and contribute to disability in advanced Parkinson’s disease (PD) patients.^1^ It is estimated that 40% of PD patients will develop these complications after 4–6 years of levodopa treatment.^2^ Deep brain stimulation (DBS) is an effective therapy to improve quality of life and to alleviate motor fluctuations by reducing off‐time and suppressing dyskinesia.^3^ DBS however, has not been shown to halt or to alter disease progression,^4^, ^5^ therefore motor fluctuations may not completely resolve or may recur despite optimization of medications and neuromodulatory therapy^6^ potentially leading to increased disability. Additionally, a subset of patients who undergo DBS surgery for PD suffer from complications of the intracranial surgery (i.e., infections, subdural hemorrhage, or lead misplacement/migration) or worsening axial motor (speech, gait, and balance) and/or non‐motor symptoms (cognitive and psychiatric).^7^ These patients may no longer be candidates for further DBS surgeries to replace the misplaced leads or to insert rescue leads. This poses a management challenge for clinicians.

In randomized controlled trials, LCIG, a continuously delivered levodopa gel by an enteral suspension infused directly into the jejunum, significantly reduced motor off‐time compared to oral levodopa therapy leading to its FDA approval in early 2015.^8^ There is, however, a paucity of data in the literature pertaining to the use, indications and long‐term efficacy of LCIG as a “rescue” therapy following DBS.^9^, ^10^

In this study, we report a series of six patients from a single tertiary referral center, who received both DBS and LCIG as interventions for optimization of their motor fluctuations. We reviewed the clinical features and discuss individual indications and outcomes of “rescue” LCIG therapy following DBS. Finally, we propose a possible clinical algorithm for inclusion of LCIG as a rescue therapy in PD DBS patients with an incomplete response to therapy.

## Case Series

We examined six patients with advanced PD and motor fluctuations treated with either globus pallidus internus (GPi) or subthalamic nucleus (STN) DBS who also had rescue LCIG therapy. The patients were recruited from a database review and were followed from January 2002 to December 2018. We retrospectively analyzed clinical data that was prospectively collected. The PD diagnoses in this cohort were in accordance with the UK Parkinson’s Disease Society Brain Bank Clinical Diagnostic Criteria^11^ and all patients were examined and managed by a fellowship trained movement disorders neurologist. The patients’ clinic‐demographical data are summarized in Table 1.

**Table 1:** Clinic‐demographical data of six patients with PD treated with DBS and LCIG therapies.

Patient	PD phenotype^a^	Sex	Age at onset of symptoms	Age at diagnosis (years)	Time from diagnosis to onset of motor fluctuations* (years)	Time from diagnosis to DBS surgery (years)^†^	Time from diagnosis to LCIG therapy (years)^‡^
1	Tremor predominant	M	42	44	9	11	18
2	Tremor predominant	M	63	63	5	5	9
3	Tremor predominant	M	46	47	11	9 and 13 (Left and Right)	14
4	Akinetic rigid	M	30	30	7	10	18
5	Akinetic rigid	M	65	66	5	5	6
6	Tremor predominant	M	70	70	6	7	10

a PD diagnoses were confirmed using the UK Parkinson’s Disease Society Brain Bank Clinical Diagnostic Criteria.

* Median time (years) from diagnosis to onset of motor fluctuations = 6.5 (5–11).

^†^ Median time (years) from diagnosis to first DBS surgery = 8 (5–11).

^‡^ Median time (years) from diagnosis to LCIG therapy = 12 (6–18).

Despite optimal medical management, disease progression with worsening off‐time and motor fluctuations was the main indication for DBS surgery. Four patients were implanted at our institution (1 bilateral GPi, 2 unilateral GPi, 1 bilateral STN). The two remaining patients were implanted with bilateral STN DBS at outside institutions. All locally implanted leads were measured using our institution’s standard protocol with high‐resolution CT scan that was acquired post lead implantation and fused with a preoperative MRI. Leads implanted at outside institutions were measured using a high‐resolution MRI. Further localization of the leads was performed using MRI morphed to a modified Schaltenbrand and Bailey atlas.^12^ A three‐dimensional representation of the lead locations relative to the targeted nucleus (GPi or STN) was performed using a locally designed software (Fig. S1)**.** Leads implanted in the posterior, ventral, and lateral aspect of GPi or dorsolateral STN were considered optimally placed if they had reasonable DBS programming thresholds.^13^

Three patients were considered to have adequately‐placed electrodes, while the remaining three had suboptimally placed electrodes. Despite initial successful management, motor fluctuations recurred in all six patients and were refractory to multiple programming attempts and medication adjustments. For patients with adequately placed unilateral leads, implantation of a contralateral lead was advised, while patients with suboptimally placed electrodes were offered surgical revision of their existing leads. A risk/benefit analysis of further DBS surgeries was conducted on all patients according to standard institutional protocol and this was performed by an interdisciplinary surgical team.^14^ Two patients were deemed to have significant cognitive decline and this risk factor precluded consideration of further neurosurgical intervention. One patient had no contraindication for bilateral lead repositioning, however elected to pursue LCIG therapy due to a hesitation about additional intracranial surgeries. Two patients had severe gastroparesis with a frequent, unpredictable response to carbidopa‐levodopa due to very poor gastrointestinal absorption. The last patient suffered an asymptomatic subdural hematoma after initial left GPi lead placement and elected not to have a contralateral lead placed for fear of further surgical complications. In all six cases, LCIG was entertained as an option for further optimization of therapy. Table 2 summarizes the DBS characteristics and the indication(s) for LCIG.

**Table 2:** UPDRS III–IV scores pre‐ and post‐DBS. Lead location and factors precluding further DBS surgeries.

Patient	UPDRS‐III scores pre‐DBS OFF‐medications	UPDRS‐III scores post‐DBS, ON‐Stimulation and OFF‐ medications	OFF‐time (scale) prior to DBS	OFF‐time (scale) after DBS	Dyskinesias duration(scale) prior to DBS	Dyskinesias duration(scale) after DBS	Lead location, comments, and factors precluding further DBS surgeries
1	25	18	N/A	N/A	N/A	N/A	Bilateral STN. Suboptimally placed leads. Cognitive decline contraindicated further DBS surgeries
2	28	19	2	1	2	0	Right GPI. Well placed lead. Cognitive decline contraindicated further DBS surgeries
3	31/34(Left DBS/Right DBS)	28/23(Left DBS/Right DBS)	N/A left 1(Right)	N/A left 1(Right)	N/A left 1(Right)	N/A left 1(Right)	Bilateral GPI. Well placed leads. Gastroparesis and unpredictable response to oral medications necessitated LCIG therapy
4	33	26	3	2	3	1	Bilateral STN. Suboptimally placed leads. Elected to pursue LCIG therapy rather than revision DBS surgery
5	29	22	1	1	4	2	Bilateral STN. Suboptimally placed DBS leads. Severe fluctuations and unpredictable response to oral levodopa necessitated LCIG therapy
6	36	23	2	1	1	0	Left GPI. Well placed lead. Developed SDH after the first DBS surgery and elected not to have further DBS surgeries

The off‐time/dyskinesia duration scale 1: 25% or less, 2: 25–50%, 3: 51–75%, 4: 76–100% of waking hours. N/A missing data. Patient 3 had two DBS surgeries 4 years apart.

The median time from diagnosis to the first DBS surgery was 8 years. All patients had expected improvement of their motor symptoms when treated with STN or GPi DBS as evidenced by UPDRS motor scores. Median off‐medication UPDRS motor scores pre‐ and post‐DBS surgery were 31 and 22.5 points, respectively, with an approximate 28% motor benefit from DBS (Table 2).

The median time from diagnosis to the use of LCIG therapy was 12 years, an average of 4 years following DBS placement. There was a 7‐point increase in median UPDRS motor scores pre‐DBS (median was 31) to median UPDRS motor scores pre‐LCIG (median was 38). Median on‐medication UPDRS motor scores did not significantly change pre‐ (Median = 25.5/IQR = 21–28) and post‐ (Median = 19.5/IQR = 15–31) LCIG therapy (*P* = 0.6). Median levodopa dose from oral carbidopa‐levodopa and levodopa equivalent from LCIG were comparable pre‐ (Median = 2300 mg/IQR = 1625–2888 mg) and post‐ (Median = 2233 mg/IQR = 1615–3054 mg) LCIG therapy (*P* = 0.752) except for one patient (Table 3) who required a higher dose of levodopa which was a strategy utilized to facilitate a taper off of rotigotine due to the development of an impulse control disorder (ICD).

**Table 3:** UPDRS III–IV scores pre‐ and post‐LCIG therapy. The UPDRS‐IV off‐time/dyskinesia duration scale 1: 25% or less, 2: 25–50%, 3: 51–75%, 4: 76–100% of waking hours.

Patient	UPDRS‐III scores Pre‐LCIG ON‐stim (OFF‐meds)	UPDRS‐III scores pre‐LCIG ON‐ Stim (ON‐meds)*	UPDRS‐III scores post‐ LCIG ON‐Stim*	OFF‐time pre‐LCIGON‐Stim^†^	OFF‐time post‐LCIG ON‐Stim^†^	Sudden OFF pre‐LCIG ON meds ON‐Stim^‡^	Sudden OFF post‐LCIG ON‐Stim^‡^	Dyskinesia disability pre‐LCIG ON‐Stim^±^	Dyskinesia disability post‐LCIG ON‐Stim^±^	Dyskinesia duration pre‐LCIG ON‐Stim^±^	Dyskinesia duration post‐LCIG ON‐Stim^±^	Levodopa dose pre‐ LCIG (mg)**	Levodopa dose equivalent from LCIG (mg)**
1	42	28	33	3	0	Y	N	2	1	4	2	3150	3216
2	33	21	19	2	1	Y	N	2	1	3	1	2750	3000
3	38	28	16	3	1	Y	Y	0	1	1	2	2800	2600
4	N/A	28	31	3	1	Y	N	3	2	4	2	1800	1600
5	28	23	14	3	1	Y	Y	2	1	3	1	1100	1866
6	39	19	20	2	0	N	N	1	2	2	4	1850	1620

Median OFF meds ON‐stim pre‐LCIG UPDRS motor scores is 38. Median OFF meds pre‐DBS UPDRS motor score (Table 2) is 31. The UPDRS‐IV dyskinesia disability scale 0: Not disabling, 1: mildly disabling, 2: moderately disabling, 3: severely disabling, 4: completely disabled.

* Median ON UPDRS motor scores pre (*Median* = 25.5/IQR = 21–28) and post (*Median* = 19.5/IQR = 15–31) LCIG therapy (*P* = 0.6) were not different. Wilcoxon signed rank test.

^†^ Median UPDRS‐IV OFF time pre (Median = 3) and post (Median = 1) LCIG therapy (ON‐Stim) were significantly different (*P* = 0.024). Wilcoxon signed rank test.

^‡^ 3 of 5 patients with unpredictable fluctuations showed improvement after LCIG therapy.

^±^ UPDRS‐IV dyskinesia disability scores pre (Median = 2) and post (Median = 1) LCIG therapy (*P* = 0.4) and dyskinesia duration scores pre (Median = 3) and post (Median = 2) LCIG therapy (*P* = 0.2) were not significantly different. Wilcoxon signed rank test.

** Median levodopa dose pre (Median = 2300 mg/IQR = 1625–2888 mg) and post (Median = 2233/IQR = 1615–3054 mg) LCIG therapy (*P* = 0.752). Wilcoxon signed rank test.

**Figure 1:**
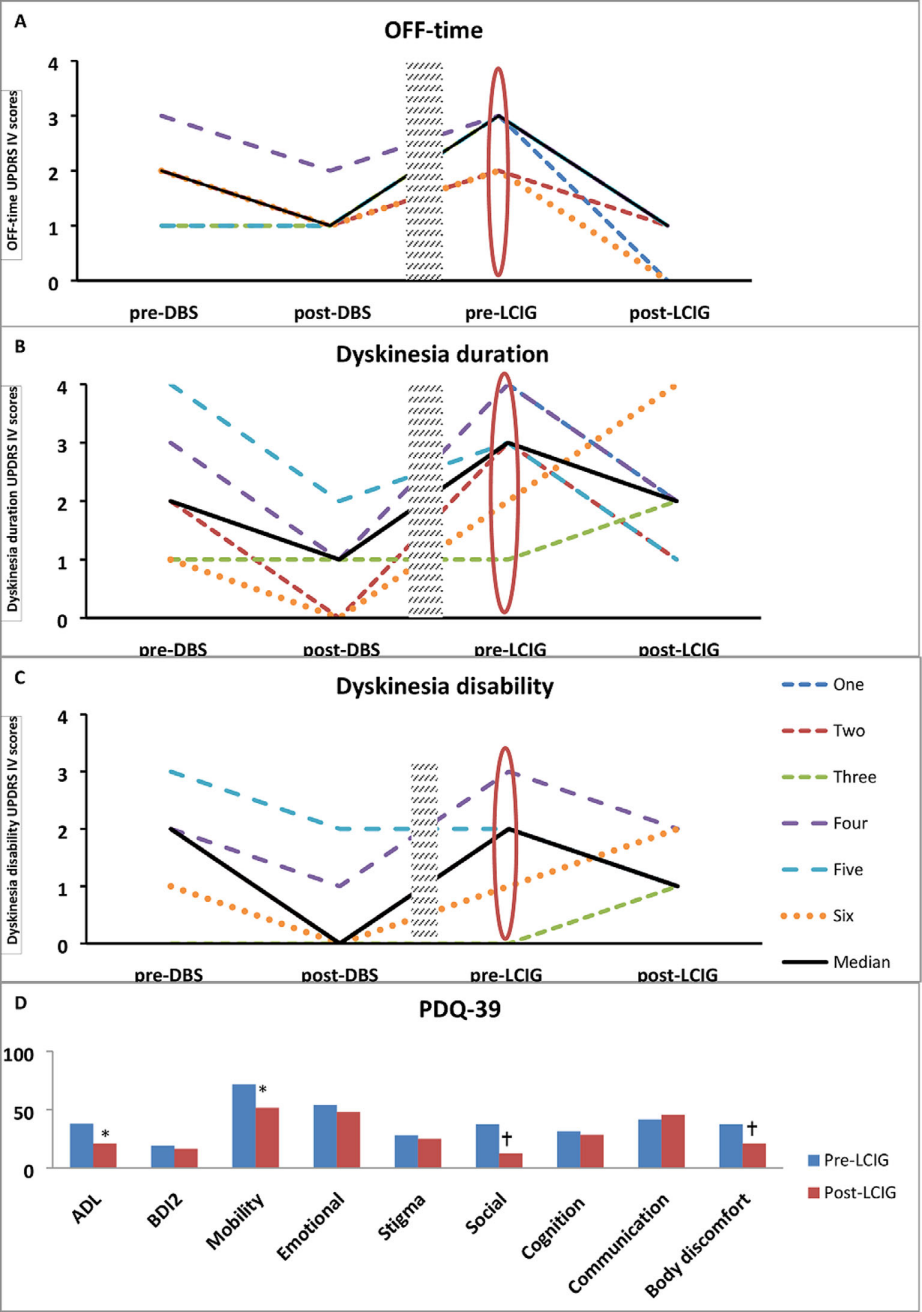
(A and B) show respectively off‐time and dyskinesia duration scales; 0: No dyskinesia, 1: 25% or less, 2: 25–50%, 3: 51–75%, 4: 76–100% of daytime hours. (A) Off‐time duration decreased in three patients after DBS surgery and uniformly decreased after LCIG therapy in all patients regardless of DBS target, laterality or accuracy of lead location (patient one had no UPDRS IV pre‐ and post‐DBS). (B) Dyskinesia duration decreased in five patients after DBS therapy but only in four patients after receiving LCIG therapy. (C) Dyskinesia disability scale 0: Not disabling, 1: mildly disabling, 2: moderately disabling, 3: severely disabling, 4: completely disabled. Dyskinesia disability decreased in four patients after DBS surgery and in three patients after LCIG therapy (patient one had no UPDRS IV pre‐ and post‐DBS). Shaded rectangle is disease progression (range 1–8 years). Red oval is the new baseline after disease progression but prior to implementation of LCIG therapy. (D) Median Quality of life scores pre‐ and post‐LCIG therapy in four patients. ADL (activity of daily living), BDI2 (Beck Depression Inventory 2). Trend toward significance (*P = 0.068) was seen in ADL and Mobility scores and in social and body discomfort scores (^†^ *P* = 0.109) using Wilcoxon Signed Rank test to compare medians.

Independent of the DBS target, laterality or accuracy of lead location, all patients had significant clinical improvement in their reported off‐time (*P* = 0.024) (Fig. 1) and three patients reported cessation of sudden off states (Fig. 1, Table 3, UPDRS‐IV scores) after LCIG therapy was implemented. Day‐time dyskinesia duration decreased in all patients after LCIG therapy except for two patients in whom the dyskinesia duration increased (Fig. 1, UPDRS‐IV scores). Four patients had quality of life scores (PDQ‐39) performed on‐medications pre‐ and post‐LCIG therapy (Fig. 1), with improvement observed across most domains, especially in activity of daily living (*P* = 0.068), mobility (*P* = 0.068)*,* social and body discomfort (*P* = 0.109).

## Discussion

We report a single‐center case series of advanced PD patients treated with GPi and STN DBS who presented with significant disease progression, as evidenced by the increase in the UPDRS motor scores from the pre‐DBS to the pre‐LCIG therapy time, and clinically significant dopaminergic off‐time. This series of six patients all benefited from DBS therapy initially, however, disease progression led to suboptimal motor symptom control (Fig. 1). Each patient reported considerable disability despite optimization of the oral therapy and DBS and this circumstance led to the consideration of LCIG. Four of our patients had contraindications or alternatively elected not to pursue further DBS surgery. Two patients developed unpredictable responses to oral medications including dose failures and worsening off‐time. LCIG proved a reasonable rescue therapy for these six patients.

In randomized DBS studies, the two main targets to treat motor complications (GPI and STN) have in general provided equivalent benefit in motor symptoms and in quality of life.^7^, ^15^ Following an institutional interdisciplinary risk/benefit assessment,^16^ choice of target for DBS is usually tailored to the individual patient’s needs. It is that in the scenario where LCIG was implemented as a DBS rescue option it was effective in treating motor complications from disease progression. It was also effective in treating motor complictions when the DBS  lead was suboptimally placed.

Regidor et al. (2017)^9^ reported 19 patients with PD who were successfully treated with bilateral STN DBS and eventually required rescue LCIG for refractory symptoms, particularly motor fluctuations and disabling dyskinesias. The authors reported a significant reduction in UPDRS motor scores after LCIG therapy (a 31‐point reduction in mean UPDRS‐III scores following LCIG therapy).^9^ In a retrospective review of seven patients with PD treated with STN, GPi or PPN DBS, Kumar et al. (2018)^10^ reported significant reduction of motor fluctuations and dyskinesia following LCIG therapy. In this series the benefit was observed regardless of brain target. Our series would potentially broaden the potential indications for LCIG as a DBS‐rescue to include optimally and sub‐optimally placed DBS leads in GPi and STN targets. Also, it would broaden potential use to unilateral DBS patients.

DBS significantly improved the baseline UPDRS motor scores across subjects. In contrast, the LCIG therapy had no significant additive effect on the on‐dopaminergic motor UPDRS (Table 2). This contrast may possibly be explained by a ceiling effect of the dopaminergic response in combination with DBS therapy. The LCIG seemed to exert its benefit on fluctuations, off‐time and quality of life. Our cohort of combined DBS‐LCIG therapy revealed improvement across most domains of PDQ‐39 with trend toward significance in activities of daily living, mobility, social, and body discomfort (Fig. 1, Table 4).^17^, ^18^

**Table 4:** Quality of life scores pre‐ and post‐LCIG therapy. Quality of life scores for patients 1, 2, 3, and 5 pre‐ and post‐LCIG therapy.

Patient	BDI2 Pre‐LCIG	BDI2 Post‐LCIG	Mobility Pre‐LCIG	Mobility Post‐LCIG	ADL Pre‐LCIG	ADL Post‐LCIG	Emotional Pre‐LCIG	Emotional Post‐LCIG	Stigma Pre‐LCIG	Stigma Post‐LCIG	Social Pre‐LCIG	Social Post‐LCIG	Cognition Pre‐LCIG	Cognition Post‐LCIG	Communication Pre‐LCIG	Communication Post‐LCIG	Body discomfort Pre‐LCIG	Body discomfort Post‐LCIG
1	26	22	85	48	63	25	63	50	25	6	50	42	69	38	67	58	50	17
2	12	15	68	55	38	29	54	46	31	44	58	17	38	38	50	58	25	25
3	25	18	75	55	38	17	54	63	50	50	0	0	19	19	33	33	75	33
5	13	6	20	8	13	0	4	4	13	0	25	8	25	19	17	17	8	0
M	19	16.5	71.5*	51.5*	38*	21*	54	48	28	25	37.5*	12.5*	31.5	28.5	41.5	45.5	37.5*	21*

M, median.

* Trends toward statistical significance, Wilcoxon signed ranked test.

**Figure 2:**
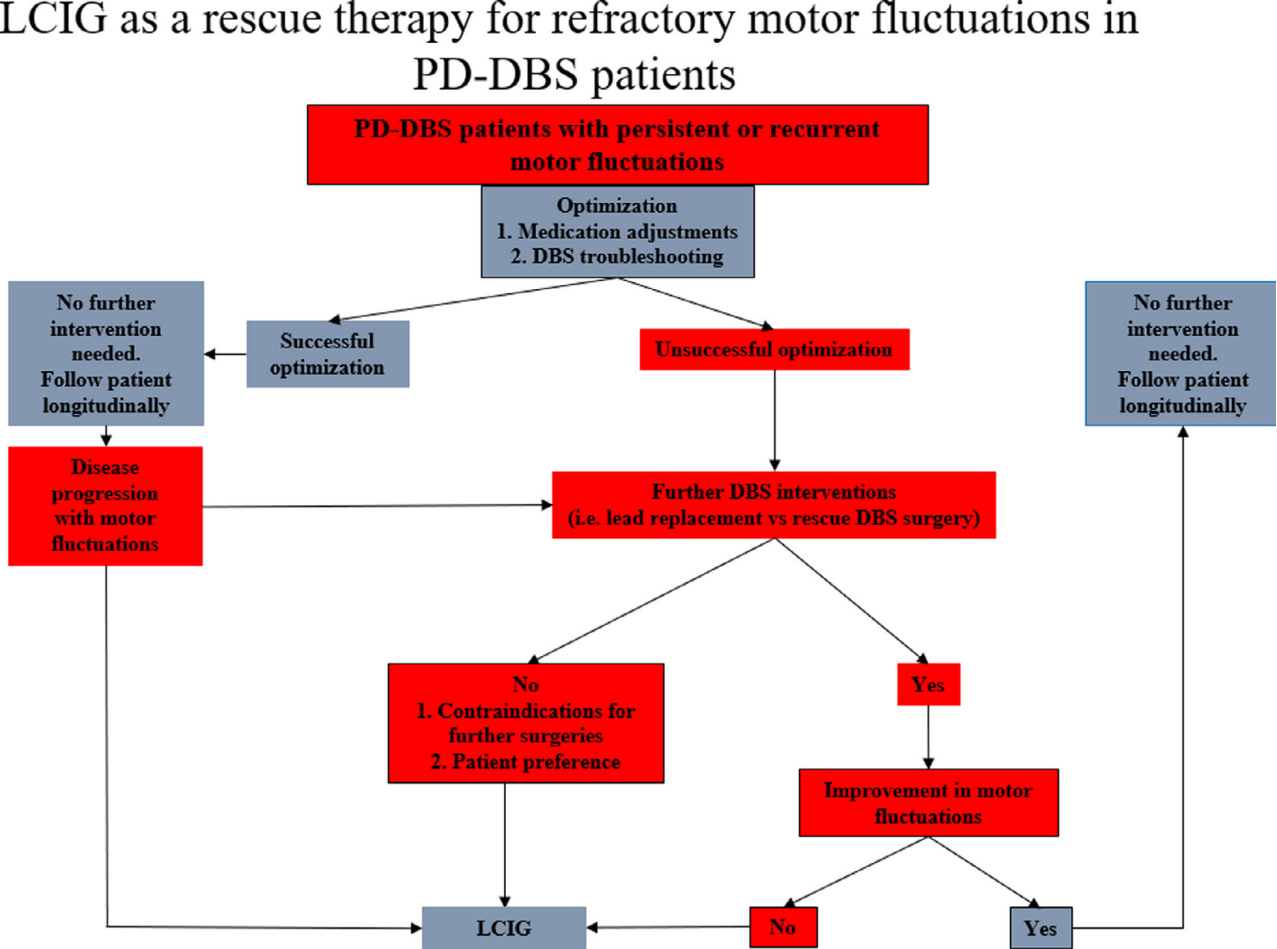
Algorithm showing the potential uses of rescue LCIG therapy in PD‐DBS patients.

LCIG can be entertained as a potential therapeutic option for the management of refractory fluctuations in PD patients following DBS. A multidisciplinary approach should be employed to determine the best treatment approach. Several factors should be carefully assessed when employing LCIG after DBS including disease progression, cognitive impairment, neuropsychiatric complications, suboptimal lead placement, patient preference, and motor fluctuations. Figure 2 offers a potential clinical algorithm that we find useful for the use of LCIG as a rescue option following DBS therapy.

In conclusion, advanced PD patients treated with DBS may continue to suffer from motor fluctuations as a result of disease progression or suboptimal DBS placement. LCIG therapy can potentially be used in select cases to “rescue” motor fluctuations and to improve quality of life. Further larger studies and data registries can help us better understand the circumstances to employ dual DBS and LCIG therapy.

## Conflict of Interest

None.

## Supporting information


**Figure S1.** A three‐dimensional representation of the lead locations relative to the targeted nucleus (GPi or STN).
