# Supplementary figures and images for: Rescue levodopa‐carbidopa intestinal gel (LCIG) therapy in Parkinson’s disease patients with suboptimal response to deep brain stimulation
Source: Ann Clin Transl Neurol. 2019 Sep 13;6(10):1989–95. doi: 10.1002/acn3.50889 (PMC6801178; doi:10.1002/acn3.50889)

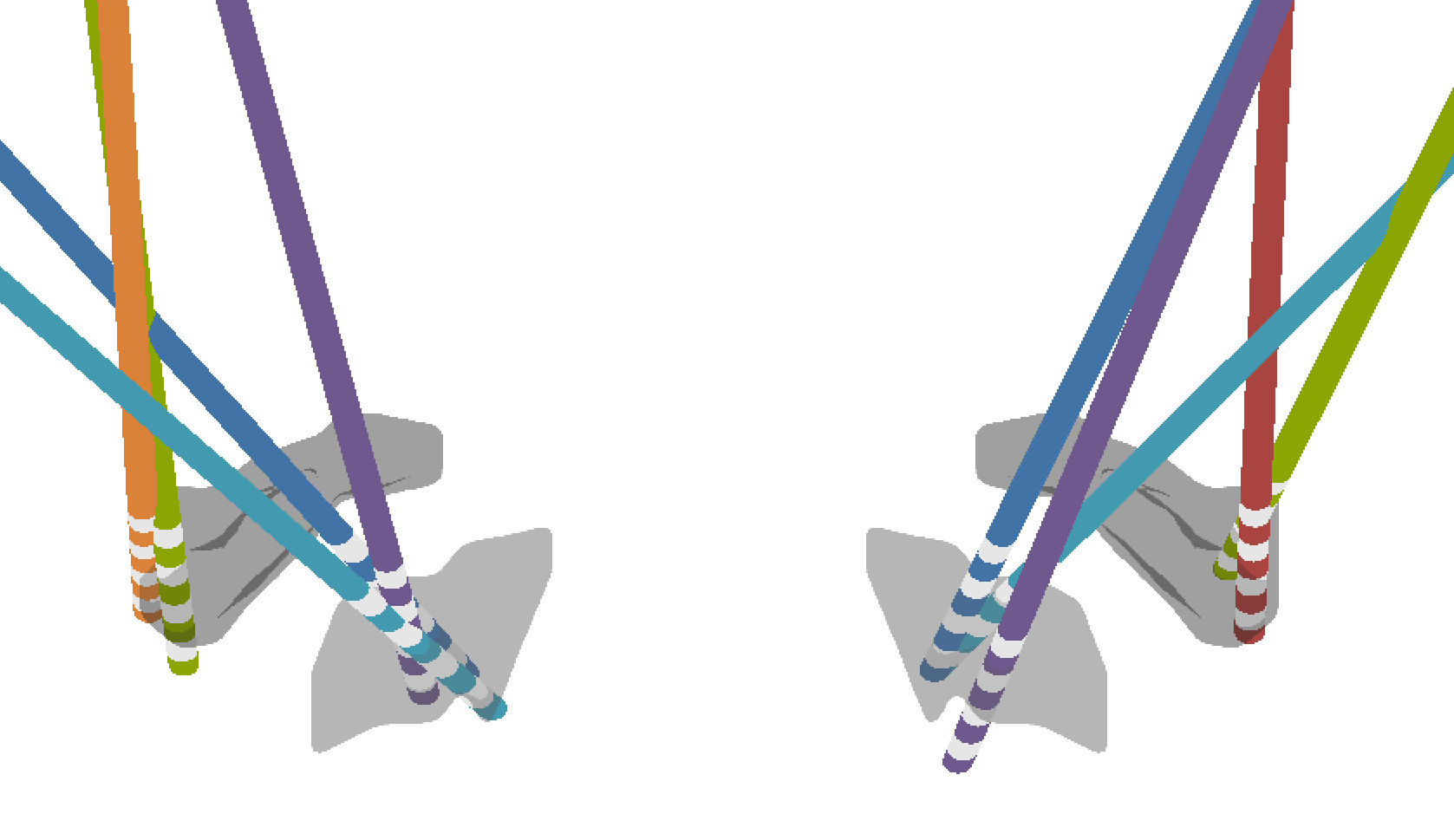

Supplement: Supplementary file 1 — Figure S1. A three‐dimensional representation of the lead locations relative to the targeted nucleus (GPi or STN). [file ACN3-6-1989-s001.png]
